# Sanitary Commission

**Published:** 1855-05

**Authors:** 


**Report of Sanitary Commssion of New Orleans on the Epidemic Yellow Fever.**

BY E. H. BARTON, M. D.

This is a work of over 500 pages, and is most interesting in its details, cogent in its reasonings, and conclusive in argument. It is the result of much labor, observation and experience, and in all respects it is one of the most interesting works which we have recently perused on the subject. The author enters into an investigation of the causes of yellow fever, and maintains that if the remedies for prevention "be seasonably applied and rigidly enforced, will not only forestall and prevent yellow fever, but from propagating should it be brought here from abroad."

He maintains that there are two causes for the yellow fever—the one, celestial or atmospheric, the other terrene or the noxious effluvia found on the surface or emanating from the soil. The last, he also maintains is most active and potent in its origination, propagation, and malignancy. But neither of these causes *alone* is competent to the production of the disease, for often and repeatedly has the sanitary condition of New Orleans been such as to furnish sufficient *pabulum* for the disease; and yet, for want of that meteorological condition, embracing solar heat, great saturation and high temperature, with a peculiar direction of the winds, all which of course are not under the control of man, no yellow fever was produced. And again, the above conditions of the atmosphere may obtain, continue, and extend and hang over a vast terrene surface whose sanitary state is good and cannot contribute one *atom* to aid them in the production of a frightful human slaughter; in that instance the celestial artillery would be compelled to retire from the occupancy of the field, without being able to prostrate a victim. It requires an *allied* force to wage successfully a pestilential war of that kind.

If then this be true it must follow that yellow fever *may* be prevented if the terrene condition be unexceptionable, and will contribute nothing to its production. It is then within human agency to prevent, provided the terrene causes can be removed, constituting a most interesting feature in the discussion of the subject, because it involves the absolute necessity of the most stringent measures, and if these stringent requirements and exactions are answered, the disease will be prevented. The corollary would be that if a city neglected the observance of the necessary sanitary measures for the preservation of health under any and all circumstances, and the meteorological conditions favored the production of yellow fever, or in other words predisposed to it, then it would extend and continue throughout such locality and until one of the two causes be withdrawn. If this position be correct, and it is well sustained by observation and experience, it would certainly be more or less under control, and the city authorities, in those localities, where they have great solar heat, saturation and exalted temperature as celestial influences, would be most criminal if they neglected the important duty of removing all sources of foul and filthy effluvia constituting elements acting in concert in the production of disease and death among the citizens for whom they are the regularly constituted sanitary guardians. Not only is this applicable to localities in which yellow fever has prevailed; but it is also true with regard to other zymotics, among which is cholera. There may exist, to a degree, an epidemic constitution abroad, and yet not to that extent or power sufficient to constitute both a predisposing and an exciting cause; in this instance it may not make itself manifest because of the absence of an exciting cause to conjoin with the foregoing. It extends still further, to another district where the sanitary condition is neglected and here it makes a sudden onslaught. This truth is made plain by every day's experience and observation during the prevalence of this disease.

It is a singular fact, as shown in this report, that yellow fever and cholera do not prevail cotemporaneously in the same district. The former requires a higher saturation as well as temperature, while the latter manifests itself in one much lower, more variable, more humid, and more modified in its capacity for evaporation, irritating "the mucous surfaces and producing in them an erethism, always a prodrome of the disease; such is just the condition attendant on epidemic influenza—the almost universal precurser of cholera."

The former seizes upon the system of organic life, leaving almost untouched the central organism, while, in the latter, the circulatory and the central systems suffer most. This is the pathological distinction set up, and accounts for the indifference first, which gradually merges into deep coma, in cholera, while in yellow fever they are sensible, mind unclouded and with the percipient faculties active to the last moments of existence.

The doctrine of the contagiousness is furiously met and completely overcome. The author says that, "if yellow fever is contagious, *it is a law of the disease*, this it must carry into all places and under all circumstances (like small-pox.) A '*contingent contagion*' is a medical misnomer, is void of a precedent, and has no parallel in the annals of science."

There are many other points in this report of much interest to which we would be glad to refer if time and space permitted. But we have already devoted more to the consideration of it than at first intended. We were so much gratified with its perusal, that we have been led on imperciptibly, step after step, occupying space and time to the neglect of other subjects of which we must speak.

